# Effectiveness of web-based mindfulness program on college students with social network addiction

**DOI:** 10.1097/MD.0000000000033022

**Published:** 2023-03-03

**Authors:** Li Yang, Lv Na, Jia Xiang Rui

**Affiliations:** a Department of Nursing, Harbin Medical University, Heilongjiang, China; b Department of Humanities and Social Sciences, Heilongjiang University of Science and Technology, Heilongjiang, China; c Department of Administration, Harbin Medical University, Heilongjiang, China.

**Keywords:** college students, emotion, mindfulness, social network addiction

## Abstract

In this study a web-based mindfulness cultivation program was implemented to investigate its effectiveness on the addiction symptoms and negative emotions of college students with social network addiction.

**Methods:**

A total of 66 students were recruited and randomly divided into the intervention group or the control group. Participants in the intervention group received a web-based mindfulness cultivation program including the group training and self-cultivating. The primary outcome was addiction level, and the secondary outcome was anxiety, depression and perceived stress. Repeated measures analysis of variance were utilized to analyze the differences between the control and intervention group over the course of the intervention and the follow-up.

**Results:**

There were significant interaction effects on addiction level (F = 39.39, *P* < .00), anxiety (F = 31.17, *P* < .00), depression (F = 37.93, *P* < .00) and perceived stress (F = 22.04, *P* < .00).

**Conclusion:**

A web-based mindfulness cultivation program could improve the addiction level and negative emotions of college students with social network addiction.

## 1. Introduction

In recent years, with the rapid development of internet technology and the wide application of tablet computers, smart phones and other mobile terminals, Weibo, Wechat, Douyin, Facebook, YouTube and other social media have gradually occupied a large amount of time in people’s daily life, becoming an important platform for people to master and check information dynamics and enhance social interactions.^[1]^ The massive popularity and use of social media in daily life certainly provides convenience, but people gradually realize that spending a lot of time on social media may cause negative effects, resulting in excessive dependence on social media and even addiction, and it can have a negative impact on users’ personal cognition, physical and mental health, and social relations.^[2,3]^ In the current age structure of Internet users, the young generation has become the main group of internet users.^[4]^ Students spend a lot of time on various social media platforms every day to share content and maintain relationships, and the risk of social media addiction is also increasing.^[5]^

Some researchers believe that the psychological and behavioral problems caused by the excessive use of social media are not enough to call it pathological addictive behavior, compared with the harm caused by internet addiction.^[6]^ However, more and more empirical research results show that when users overly on or use social media in problematic ways, they are prone to sleep disorders, decreased academic performance, decreased work efficiency and other behaviors, and negative impacts on personal physical and mental health.^[7]^ Social media addiction is used interchangeably to refer to the phenomenon of maladaptive social media use characterized by either addictive-like symptoms and/or reduced self-regulation.^[8]^

Empirical evidence suggests that compulsive social media addiction is becoming a growing mental health problem, especially among adolescents, which is closely related to students’ academic performance,^[9]^ sleep disorder,^[10]^ anxiety,^[11]^ and depression.^[12]^

Many attempts have been made in improving internet addiction and smartphone addiction; however, although the phenomenon of social media addiction has become increasingly serious in recent years, there is still a lack of targeted interventions.^[13,14]^ Mindfulness is an “Awareness generated through intentional and nonjudgmental attention to the present moment.”^[15]^ Studies have shown that mindfulness training can be effective in alleviating physical and mental health problems and has been widely used in different populations.^[16,17]^ Khanna et al proposed that the state of “mindfulness deficit” and its associated negative emotions and life events are 1 of the reasons for individual addictive behaviors, including escapism, automatic thinking, emotional reaction, and social isolation.^[8]^ Mindfulness cultivation is to integrate the concept, method and technology of mindfulness into all parts of life that enhance individual self-regulation, acquisition acceptance and nonjudgment, increase prosocial behavior, and then cultivate a positive attitude.^[15]^ However, few studies have been conducted so far to look specifically into the effects of mindfulness on social media addiction. Thus, this study developed a web-based mindfulness cultivation scheme and applied it to the college students with social media addiction to investigate its effectiveness on addiction symptoms and negative emotions in the short-term and long-term.

## 2. Materials & methods

### 2.1. Study design

The study utilized a signal-blind, 2-arm randomized control trial to evaluate the efficacy of a web-based mindfulness cultivation on addiction symptoms and negative emotions of college students with social media addiction.

### 2.2. Sample size

The sample size was estimated using G*Power3.1 software. According to analysis of variance of repeated measurement data, effect size was set as medium 0.25, test level (α) was 0.05, statistical test power (1-β) was 0.8, number of groups was 2, number of tests was 3, and correlation coefficient of repeated measurement was 0.5. Preliminary estimates of the total sample size required were 52. Allowing for the 20% drop out rate, finally we recruited 66 participants.

### 2.3. Participants and recruitment

From August to September 2020, participants of “Cultivating College Students’ Mindfulness” were recruited through an online propaganda in 2 universities.

**Figure 1.**
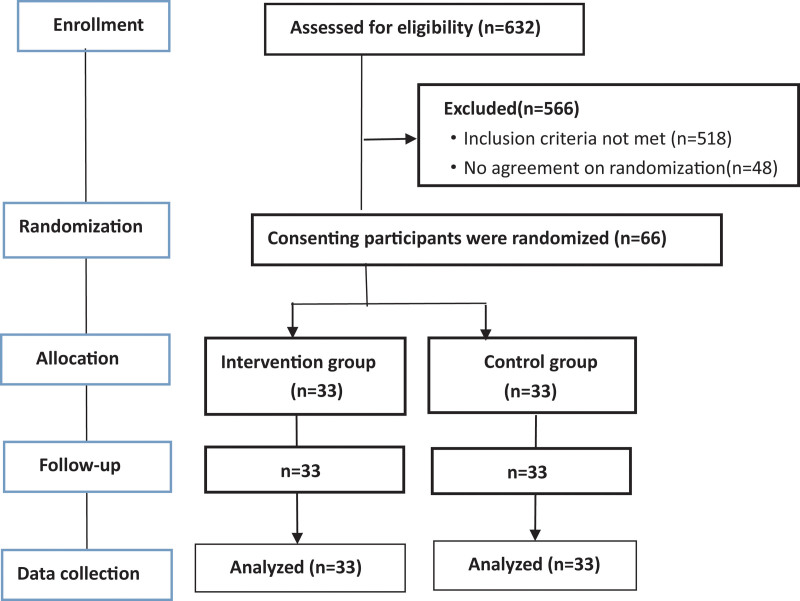
Study flow diagram: Enrollment to analysis.

The requirements and procedure were delivered through the recruitment, all participants who were interested in the program submitted the application form and were interviewed by an assistant to evaluate whether they met the inclusion criteria:  > 18 years old; diagnosed with social media addiction; no related treatment experience (including yoga, Taiji Chuan, etc); willing to participate. Participants who had major mental disease (such as schizophrenia and bipolar disorder) were excluded. Finally, 66 students were recruited in the study and randomly assigned to intervention group or control group by the random number table (seen in Fig. 1). All subjects signed electronic informed consent. This study was approved by the Ethics Committee of the Department of Harbin Medical University which complies with the Helsinki Declaration.

### 2.4. Interventions

#### 2.4.1. Intervention group.

##### 2.4.1.1. Preparation before intervention.

An intervention implementation team was established, including 2 assistants and 1 psychologist who was skilled in mindfulness cultivation and had the mindfulness training qualification. Assistant A was responsible for dealing with the network technical problems reported by students and keeping attendance records of students. Assistant B was in charge of managing the training group to monitor students to practice independently every day. The psychologist was responsible for implementing the web-based mindfulness cultivation course to the intervention group members.

##### 2.4.1.2. The implement of the web-based mindfulness cultivation program

Compared with traditional mindfulness intervention, this program made 2 adjustments: first, it brought mindfulness training techniques to life and cultivated the mindfulness anytime and anywhere. The main goal of the program was to let participants grasp the core of mindfulness and apply it to life. Second, it shortened the duration of a single activity, standardized the guidance language of mindfulness training, and emphasized daily continuous training, which is more suitable for college students to participate in.

The process of cultivating mindfulness follows the process of “concept of mindfulness - experience of mindfulness - practice of mindfulness - sharing of mindfulness.” Each group intervention was carried out on Tencent conference software, and cameras and microphones needed to be turned on during the whole process. Continuous practice was essential to mindfulness cultivation, so in addition to group intervention, participants were encouraged to continue to practice through mini-program clocking every day. The group web-based intervention included 8 courses and each course was 50 to 60 minutes. The specific content of each course is shown in Table 1.

**Table 1:** The details of each session.

Wk	Theme	Content
1	Introduction	Introduce the background and theoretical basis of mindfulness cultivation (Formal training course arrangement, informal training method, time requirement and regular report). Subjects were given videos and audio files.
2	Basic skills	Mindfulness breathing & Mindfulness meditation: Introduce the 2 basic mindfulness skills; Practice to get the skills; Homework
3	Listening	Mindfulness Listening: Explain the core of mindfulness listening and teach the skills to be mindfulness. Practice and Homework.
4	Walking	Mindfulness Walking: Explain the core of mindfulness walking and teach the skills to be mindfulness. Practice and Homework.
5	Seeing	Mindfulness Seeing: Explain the core of mindfulness seeing and teach the skills to be mindfulness. Practice and Homework.
6	Smelling	Mindfulness Smelling: Explain the core of mindfulness smelling and teach the skills to be mindfulness. Practice and Homework.
7	Eating	Mindfulness Eating: Explain the core of mindfulness eating and teach the skills to be mindfulness. Practice and Homework.
8	Keeping	Group members share their experience and confusion of informal training. Subjects were required to continue informal training and integrated mindfulness into daily life.

#### 2.4.2. Wait-list control group.

Participants in the wait-list control group continued with their lives as usual. After the intervention completed, they received the same program according to their needs.

### 2.5. Measurement

All participants needed to finish the electronic questionnaires 3 times at T1-before intervention, T2-after intervention, T3-half-year after intervention.

#### 1.2.5. *The social media disorder scale (SMD*).

This scale has 9 dichotomous questions (i.e., yes or no). Individuals are diagnosed with social media addiction when they meet 5 or more of these 9 criteria. The scale is widely used in China and has good reliability and validity.^[18]^ In the current study, the Cronbach alpha was 0.79.

#### 2.2.5. *Self-rating anxiety scale (SAS) and self-rating depression scale (SDS).*

The SAS^[19]^ and SDS^[20]^ were used to evaluate anxiety and depression level of the participants, respectively. They both consist of twenty items and each item is scored by Likert 1 to 4. Higher scores indicate higher anxiety or depression level. A score of 50 to 59 indicates mild anxiety or depression, 60 to 69 moderate anxiety or moderate depression, and above 70 relatively severe anxiety and major depression. The 2 scales are widely used in China and have good reliability and validity.^[21]^ The Cronbach’s alpha value in this study were found to be 0.80 and 0.81, respectively.

#### 3.2.5. Perceived stress scale (PSS).

This scale was translated by Yang et al,^[22]^ which consists of 14 items rated on a 5-point scale (0–4), with a total score ranging from 0 to 56. Higher scores indicated higher perceived stress level. In this study, the Cronbach’s alpha was 0.79.

### 2.6. Statistical analysis

SPSS25.0 software was used for quantitative data analysis, and repeated measure analysis of variance was used to compare the scores of addiction symptoms, depression, anxiety and perceived stress at 3 time points. The difference of these variables were compared between 2 groups at each time by *t* test. *P* < .05 was considered a statistically significant difference.

## 3. Results

### 3.1. The Demographic information

The average age of the participants was 20.14 ± 1.81 years, with a range of 17 to 24 years. Table 2 displays the characteristics of the 2 groups.

**Table 2:** Comparison of demographic characteristics of 2 groups (n = 66).

	Intervention group (n = 33)	Control group (n = 33)	*t* or *χ*^2^	*P*
**Age (mean ± SD**)	20.52 ± 1.66	20.39 ± 1.58	0.30†	.76
**Grade**			0.34‡	.85
Freshman	9	7		
Sophomore	15	16		
Junior	9	10		
**Residence**			0.29‡	.59
Urban	22	24		
Rural	11	9		
**Gender**			0.31‡	.58
Men	10	8		
Women	23	25		
**Only-Child or not**			0.60‡	.44
Yes	23	20		
No	10	13		

† Present *t* test.

‡ Present *χ*^2^ test.

### 3.2. Efficacy

#### 3.2.1. Effects of intervention and time factors on addiction symptoms, anxiety, depression, and perceived stress.

There were significant interaction effects between group and time in total score and each dimension of SMD, SAS, SDS, and PSS (*P* < .05). See Table 3.

**Table 3:** ANOVA effect values of SMD, SAS, SDS, and PSS of the 2 groups before and after intervention.

Items	Group effect		Time effect		Group × Time effect	
	*F*	*P*	*F*	*P*	*F*	*P*
**SMD**	31.7	<.00	72.07	<.00	39.39	<.00
**SAS**	4.61	.04	43.31	<.00	31.17	<.00
**SDS**	4.42	.04	46.03	<.00	37.93	<.00
**PSS**	5.43	.03	8.78	<.00	22.04	<.00

ANOVA = analysis of variance, PSS = perceived stress scale, SAS = self-rating anxiety scale, SDS = self-rating depression scale, SMD = social media disorder.

#### 3.2.2. Compare of SMD, SAS, SDS, and PSS scores before and after intervention between 2 groups.

The results of Greenhouse-Geisser correction and repeated measure variance analysis showed that there was no significant difference in the scores of SMD, SAS, SDS, and PSS between the 2 groups before intervention (all *P* > .05). After intervention, the scores of SMD, SAS, SDS, and PSS in the intervention group were significantly different from control group at T2 and T3 (all *P* < .05), while the scores of SMD, SAS, SDS and PSS in intervention group were significantly lower than control group at T2 and T3. See Table 4.

**Table 4:** Comparison of SMD, SAS, SDS, PSS between 2 groups before and after intervention (score, x ± s).

Items	Group	T1-baseline	T2-after intervention	T3-6 months after intervention	*F*	*P*
**SMD**	Intervention	5.42 ± 0.61	4.67 ± 0.60	4.01 ± 0.50	51.24	<.00
	Control	5.51 ± 0.62	5.36 ± 0.65	5.30 ± 0.85	1.03	.36
	*t*	−0.51	−4.21	−8.83		
	*P*	.61	<.05	<.05		
**SAS**	Intervention	52.39 ± 5.77	47.00 ± 4.71	45.51 ± 4.29	53.06	<.00
**1.19**	Control	51.33 ± 6.57	51.61 ± 6.98	50.78 ± 6.73	2.27	.12
	*t*	−0.71	3.38	3.70		
	*P*	.48	<.05	<.05		
**SDS**	Intervention	52.52 ± 5.90	48.48 ± 4.72	46.91 ± 4.25	83.41	.02
	Control	52.36 ± 6.07	51.81 ± 5.92	52.21 ± 6.33	1.29	.29
	*t*	−0.10	2.58	3.82		
	*P*	.92	.02	<.00		
**PSS**	Intervention	30.85 ± 6.01	28.61 ± 4.83	27.24 ± 4.64	40.82	<.00
	Control	31.21 ± 5.08	31.36 ± 5.11	32.33 ± 5.19	1.62	.214
	*t*	−0.27	2.41	4.23		
	*P*	.78	.02	<.00		
	*P*	.17	<.05	<.00		

PSS = perceived stress scale, SAS = self-rating anxiety scale, SDS = self-rating depression scale, SMD = social media disorder.

## 4. Discussion

To the best of our knowledge, this is the first study to investigate the effectiveness of an online mindfulness cultivation program on college students with social media addiction in China which has yielded significant results. The results of this study show that the online mindfulness cultivation program can improve the addiction symptoms and decrease the negative emotions such as anxiety, depression and perceived stress of college students. Surprisingly, the effectiveness lasted for a half-year during the follow-up. The intervention content of this study focused on the cultivation of mindfulness instead of the short-term mindfulness skills training. The aim of the program was to let participants consistently incorporate mindfulness into their life and study.

The results showed that the social addiction level among college students in the intervention group were significantly decreased at T2 and T3, indicating that the mindfulness program was effective in improving the social media addiction among college students. Surbhi & Jeffrey^[23]^ believe that addiction is caused by a series of emotional reactions and social problems involved in the state of “mindfulness deficit,” and the empty feeling will lead individuals to satisfy themselves through alcohol or other substances. And mindfulness training methods can improve the level of individual mindfulness and remove the individual “lack of mindfulness state.”^[24]^ Many studies reported similar results in a study of mindfulness applied in addiction individuals such as food addiction^[25]^ and substance abuse.^[26]^ Mindfulness training helps individuals pay more attention to their current state, not react to addiction cues, not judge, and not only weakened the subjective feelings of bad emotions, but also changed the way of reaction, and finally improved the addictive behavior.^[27]^ Therefore, most of the individuals in the group who had undergone mindfulness cultivation can correctly treat social media, and basically realize the awareness of information but do not respond immediately. On the one hand, the improvement of the mindfulness level of individuals is due to the fact that they have never been exposed to the intervention of relevant treatment means, and on the other hand, they have the willingness to change their dependence on social media.

In recent years, researchers in the field of addiction have become increasingly aware of the close relationship between substance abuse and stress and negative emotions^[28,29]^ and studies have found that substance abuse is often seen as an individual’s maladaptive response to stress.^[27]^ The results showed that the college students with social media addiction have a high level of perceived stress and negative emotions. The results showed that the detection rate of anxiety and depression among college students were 61% and 69%, indicating that the mental health status of college students with social media addiction were at a poor level. This is similar to related studies.^[30,31]^ Through the mindfulness cultivation program, the anxiety and depression rate in the intervention group were significantly decrease to 24% and 30%, respectively. Moreover, the perceived stress decreased from 30.85 ± 6.01 at baseline to 28.61 ± 4.83 at T2 and 27.24 ± 4.64 at T3. Mindfulness training guides students to look at current experiences from a “nonjudgmental” perspective and be open to them, whether they are pleasant or not.^[32]^ This way can help students timely regulate bad emotions and improve the ability of emotion management. In addition to reducing negative emotions and eliminating mental diseases, mindfulness training can better help individuals establish positive cognition, discover and develop their own potential, and achieve overall healthy development of body and mind.^[33]^ In addition, compared with traditional infusion education, when students master the method of mindfulness training, they can conduct mindfulness training more autonomously, further improving their mental health level.^[34]^

Importantly, the effectiveness of the short-term online mindfulness cultivation on addiction symptoms and negative emotions lasts for 6 months. Mindfulness cultivation in this study included both formal and informal mindfulness practices. Informal mindfulness practice, learned from the Chinese excellent traditional culture of “mindfulness” nutrient, revolves around the psychological and behavior, such as “eat slowly,” “eat & sleep silence,” “knowledge-action unity,” etc, to experience and comprehension, practice and consolidation. Formal mindfulness practice involves low-intensity intervention training such as “recognition of automatic thinking” and “mindful breathing” to develop an attitude and capacity for attention and acceptance. Through the continuous cultivation in daily life, the participants would be open, clear and calm in the face of external things thus leading to the inner neutrality that was not affected by deviation.^[35]^ This study cultivated flexibility in dealing with problems in daily life, and put down “ego obsession” (automatic thinking). Mindfulness, with an open and receptive attitude, was more flexible and reasonable in the allocation of attention function and increases the resilience of individual emotional response.^[36]^ After the cultivation of mindfulness, especially the informal mindfulness experience in daily life, participants have certain self-awareness and response to the habitual and irrational thinking mode. They integrated “present awareness” into life to achieve “no choice awareness.” Each course has a strong structure, and each activity was divided into small dimensions which were easier for college students to learn and apply. The intervention content of this study focused on attention to and acceptance of mindfulness and acquire a mindfulness life.

### 4.1. Strengths and limitations

Mindfulness cultivation included but was not limited to mindfulness training, which integrated the concept, method and technology of mindfulness into all parts of life. Through the approach of mindfulness cultivation, individuals could enhance self-regulation, acquire acceptance and nonjudgment, and then cultivate a positive attitude. The results of this study proved that mindfulness cultivation could be used as a feasible and operational self-training method to improve the social media addiction and mental health of college students. However, there were still some limitations in this study. Firstly, the participants were recruited from 1 province and the results may not be generalized to the whole population. In addition, the questionnaires were all self-reported measures. In future studies, more objective, behavioral or physiological measures should be used to evaluate various variables which will further enhance the validity of intervention.

## 5. Conclusion

This study constructed a mindfulness cultivation program integrated relevant skills into daily life, which decreased the addiction symptoms and negative effects such as anxiety, depression, and perceived stress of college students with social media addiction. This program provided a flexible and effective intervention for college students with social media addiction. In the future, studies may focus on the mechanism of how mindfulness affects social media addiction. The intervention research of mindfulness on decision-making disorders and biases in different addicts is an important aspect worth exploring.

## Acknowledgments

All authors were grateful for all the participants and all student counselors in this study for their cooperation.

## Author contributions

**Conceptualization:** Li Yang, Lv Na, Jia Xiang Rui.

**Data curation:** Li Yang, Jia Xiang Rui.

**Formal analysis:** Lv Na.

**Funding acquisition:** Li Yang.

**Investigation:** Li Yang, Lv Na, Jia Xiang Rui.

**Methodology:** Lv Na, Jia Xiang Rui.

**Resources:** Lv Na.

**Supervision:** Li Yang.

**Validation:** Lv Na, Jia Xiang Rui.

**Writing – original draft:** Li Yang, Lv Na, Jia Xiang Rui.

**Writing – review & editing:** Li Yang, Lv Na, Jia Xiang Rui.
